# Lethal and Sublethal Effects of Three Microbial Biocontrol Agents on *Spodoptera litura* and Its Natural Predator *Rhynocoris kumarii*

**DOI:** 10.3390/insects9030101

**Published:** 2018-08-14

**Authors:** Kitherian Sahayaraj, Natarajan Subash, Robert W. Allingham, Vivek Kumar, Pasco B. Avery, Lucky K. Mehra, Cindy L. McKenzie, Lance S. Osborne

**Affiliations:** 1 Crop Protection Research Centre, St. Xavier’s College (Autonomous), Manonmaniam Sundaranar University, Palayamkottai 627002, India; nsubash@gmail.com; 2 Fruinlaan 43, 5624 DA Eindhoven, The Netherlands; allingham.rob@gmail.com; 3 Mid-Florida Research and Education Center, Institute of Food and Agricultural Sciences, University of Florida, 2725, South Binion Road, Apopka, FL 32703, USA; vivekiari@ufl.edu (V.K.); lsosborn@ufl.edu (L.S.O.); 4 United States Department of Agriculture, Agricultural Research Services, 2001 South Rock Road, Fort Pierce, FL 34945, USA; cindy.mckenzie@ars.usda.gov; 5 Indian River Research and Education Center, Institute of Food and Agricultural Sciences, University of Florida, 2199 South Rock Road, Fort Pierce, FL 34945, USA; pbavery@ufl.edu; 6 Department of Plant Pathology, Kansas State University, 1712 Claflin Rd., 4024 Throckmorton Center, Manhattan, KS 66506, USA; luckymehra@ksu.edu

**Keywords:** *Metarizhium anisopliae*, *Pseudomonas fluorescens*, integrated pest management, field crop pest, nucleopolyhedrovirus, entomopathogens

## Abstract

Entomopathogenic microbes such as *Spodoptera litura* nucleopolyhedrovirus (SpltNPV), *Metarhizium anisopliae*, and *Pseudomonas fluorescens* are biological agents used for the control of multiple arthropod pests. The objective of this study was to assess their effects on the biological parameters of *Spodoptera litura* (Lepidoptera: Noctuidae) larvae, and its natural reduviid predator *Rhynocoris kumarii* (Hemiptera: Reduviidae) under laboratory conditions. Results suggested that *P. fluorescens* reduced the food consumption index, relative growth rate, approximate digestibility, the efficiency of conversion of ingested food, and the efficiency of conversion of digested food of *S. litura* third instar larvae compared to prey infected with *M. anisopliae* and SpltNPV. Both SpltNPV and *M. anisopliae* caused similar mortality of *S. litura* life stages after 96 h of observation. To observe the effect of an infected prey diet on predator behavior, infected *S. litura* larvae were offered to the third, fourth, and fifth instar nymphs of *R. kumarii*, and their prey handling time, predation rate (number/day/predator), developmental period, and the survival rate was recorded. When the life stages of *R. kumarii* were offered entomopathogen-infected *S. litura* larvae, their predation rate was comparable to or higher than the untreated control. The juvenile predator, after feeding on *P. fluorescens-*infected *S. litura* larvae, had a significantly longer developmental period (2–4 days) compared to those fed on larvae infected with other microbial control agents. However, feeding on *P. fluorescens* alone did not affect the predator nymphal survival rate or the adult sex ratio. Although three entomopathogens had some degree of effect on the biological parameters of *R. kumarii*, the outcome of this study suggests that integration of reduviids with the tested entomopathogens are a compatible and potentially effective strategy for the management of *S. litura* populations. However promising, this combined strategy needs to be tested under field conditions to confirm the laboratory findings.

## 1. Introduction

The cutworm, *Spodoptera litura* (Fab.) (Lepidoptera: Noctuidae), known to attack over 120 plant species among vegetables, fruits, and ornamentals worldwide, is a serious pest of field crops in India [1]. It is widespread throughout Indian states and known for its economic impact in cotton, tobacco, and soybean production [2], and in groundnut, it is known to cause yield losses of 35–55% [3]. Due to the economic importance of many of its plant hosts, insecticide applications are considered the primary method for managing *S. litura* during the crop growing seasons [3].The indiscriminate and large-scale use of synthetic chemical pesticides to manage this pest has resulted in ecological imbalances, especially among natural enemies, as well as toxic effects on other living organisms, including humans [4]. There are also reports that *S. litura* has developed resistance against many synthetic insecticides [5,6,7], which may have direct consequences on the agricultural sectors in the developing nations with limited resources. Thus, in order to offer our growers alternatives to harmful synthetic chemical pesticides, it is imperative to explore eco-friendly (ecological + economical) pest management strategies utilizing biological control agents that could provide similar efficacy against this pest.

In the past, several researchers endorsed the utility of naturally occurring entomopathogens such as *Spodoptera litura* nucleopolyhedrovirus (SpltNPV), *Metarizhium anisopliae* (Metsch.) Sorokin (=*flavoviride*) Gams and Rozsypal var. *acridum*, and *Pseudomonas fluorescens* (Trevisan) Migula [8,9,10,11,12,13,14] for management of field crop pests. In India, under field conditions, nuclear polyhedrosis virus (NPV-S) reduced *S. litura* populations in cabbage [15] and soybean [16]. In Assam, following the application of *M. anisopliae* (strains MTCC-4034 and 4645), termite populations were reduced and tea leaf yield was improved [17]. There is abundant literature available for the utilization of SpltNPV and *M. anisopliae* [17,18,19,20,21,22,23,24,25,26], but very little published information concerning the efficacy of using *P. fluorescens* against field crop pests. This pseudomonas bacteria were shown to be effective against certain arthropod pests [27], including tobacco hornworm, *Manduca sexta* L. [12], and the spider mites *Oligonychus coffeae* Nietner and *O. afrasiaticus* Mc Gregor [28,29].

Hunter reduviids (Reduviidae) are generalist predators of multiple species of Lepidoptera, Coleoptera, Hemiptera, Orthoptera, and Isoptera [30]. These predators are commonly found in the field of many agronomic crops, and they play a major role in the natural suppression of several pests including *Helicoverpa armigera* Hubner, *Creontiades dilutus* (Stål), *Spodoptera litura* (Fab.), *Dysdercus cingulatus* (Fab.), *Phenacoccus solenopsis* (Tinsley), *Aphis gossypii* (Glover), *Euproctis mollifera* (Thunberg), and *Anomis flava* (Fabricius) [31,32,33,34,35]. In India, among several reduviid species, *Rhynocoris kumarii* Ambrose and Livingstone has been reported to feed on economically important agricultural arthropod pests under controlled and field conditions [34,35,36,37,38,39,40,41]. In cotton agroecosystems, augmentative releases of this predator have been recommended for the management of *S. litura*, *H. armigera*, and *E. mollifera* [37,40], whereas in okra, the reduviids have been found effective against *H. armigera* and *A. flava* [38]. 

Authors have explored the multitrophic interactions and studied the effects of entomopathogenic microbes against nontarget organisms including natural enemies [42,43]. However, the interaction of such microbial biopesticides with the predatory reduviids, such as *R. kumarii*, is still understudied. Sajap et al. [44] evaluated the impact of nuclear polyhedrosis virus (NPV) on the hemipteran predator, *Sycanus leucomesus* Walker, under laboratory conditions and found that the NPV treatment reduced nymphal survival rate, adult longevity, and fecundity. Similarly, Abbas et al. [45] assessed the impact of NPV-infected *Anticarsia gemmatalis* (Hübner) against the predatory stinkbug, *Podisus maculiventris* (Say), and found that they were compatible when applied in combination. In India, the majority of research has focused on assessing the impact of synthetic broad-spectrum insecticides against *R. kumarii* [46], and the side-effects of microbes and insecticides on other reduviid predators [47,48,49,50]. Due to the lack of information on the interaction of potential compatibility between microbial biocontrol agents of *S. litura* and their reduviid predators, their possible integration in various pest management programs has not been implemented. Thus, in this study, we determined the effect of the microbial biological control agents SpltNPV, *M. anisopliae*, and *P. fluorescens* on biological parameters including feeding time, number and amount of food consumed, nymphal development, and survival of *R. kumarii* when exposed to infected *S. litura* larvae.

## 2. Materials and Methods

### 2.1. Collection and Rearing of Insects

Life stages of *R. kumarii* were collected from the scrub jungles which bordered the cotton agroecosystems of the Marunthuvazh Malai (8.1323° N, 77.5118° E), Kanyakumari district, Tamil Nadu, India, and were maintained under laboratory conditions at 30 ± 1 °C and 60–70% relative humidity (RH) under a 11 h light (L): 13 h dark (D) photoperiod for three generations. They were reared on third, fourth, and fifth stadium larvae of *S. litura*, and were kept in round plastic boxes (6.5 cm × 16.0 cm; Sun Shine, Tirunelveli, India). Filter paper was placed at the bottom of the box, and chart paper folded into a zigzag pattern (10 × 15 cm) was placed on top of it for nesting of the predator. Newly hatched nymphs (≥24 h) were used for experimental purposes.

Life stages of *S. litura* were collected from groundnut, castor, and okra fields in the Tirunelveli district, Tamil Nadu, India. The larvae were reared in the laboratory in plastic containers (2 L capacity, 20 cm diameter) on fresh castor bean leaves. The castor plants were grown in the crop screen house. Newly emerged third instar larvae (>6 h) weighing 0.3–0.35 g were used for the experiments to avoid any ambiguity due to biomass. Cotton (variety SVPR2) plants were seeded in plastic trays in a screen house under natural lighting conditions (30 ± 2 °C; 55–67% RH; 11:13 h L:D photoperiod). One week after germination, the cotton seedlings were individually transplanted into 1 L mud pots. The seedlings were fertilized with NPK fertilizer (80:40:40 of N, P_2_O_5_, K_2_O) weekly (5–10 g/pot) and watered as needed. Four-week old cotton plants were used in all experiments. The experimental setup was maintained under laboratory conditions as described above. 

### 2.2. Microbial Biocontrol Agents and Bioassay for Assessment

Stock culture of *M. anisopliae* (1.2 × 10^11^ conidia/mL) was provided by the Crop Protection Research Centre, and both SpltNPV (1 × 10^8^ polyhedral occlusion bodies) and *P. fluorescens* (1 mL of the broth = 2 × 10^8^ colony-forming units/gram) were purchased from the district agricultural office in Palayamkottai as a technical formulated liquid. Viability for all the microbial formulations was >95%. The microbial product suspensions were sprayed with a hand sprayer (Shradha Plastic, Mumbai, India) on both sides of the cotton (variety SVPR2) leaf surfaces on the plants until runoff. The treated leaves were left to air-dry naturally in the laboratory. Leaves sprayed with sterilized tap water were used as a control treatment. Treated leaves were fed to third instar *S. litura* larvae as needed. They were kept in plastic containers (6.5 cm diameter and 9 cm height) and covered with muslin cloth (Ram Textiles, Erode, Tamil Nadu, India). The larvae were allowed to feed on cotton leaves for 96 h continuously under the laboratory conditions described above. Mortality was recorded 48, 72, and 96 h after treatment. There were 10 replicate leaves per treatment and untreated controls were used for each treatment.

### 2.3. Determining the Feeding Deterrence Index

Feeding deterrence index (FDI) was determined using a leaf disk no-choice assay. Disks (8 cm^2^; mean fresh weight = 300–320 mg) were cut from cotton leaves and dipped to insure coverage on both sides for each of the following treatments: (1) 5.0 mL of the *M. anisopliae* suspension, which was emulsified with Tween 80^®^ (0.1%) solution as a carrier, (2) technical grade liquid formulation of SpltNPV, and (3) technical grade liquid formulation of *P. fluorescens.* The products used for the treatments of SpltNPV and *P. fluorescens* were used without an adjuvant because there was a carrier present in the formulation. Control cotton leaves were dipped in 5.0 mL water (99 mL distilled water + 1 mL Tween 80^®^ (0.1%) solution. The leaf disks were dried at room temperature, and newly emerged third instar *S. litura* larvae were removed from the stock culture and starved for 4 h before testing. These preweighed (in mg) larvae were placed into Petri dishes (9.5 cm × 1.5 cm) containing either a microbial treatment or surfactant (0.1% Tween 80^®^) solution on a pretreated cotton leaf disk. Sterilized tap water and a small piece of moistened cotton was placed inside the Petri dishes to protect the leaves from desiccation. Ten replicate dish chambers (closed with lid) containing the leaf disk and the larva were used to assess the FDI values for each microbial treatment and control. Larvae were allowed to feed continuously for four days. Daily, the amount of food consumed by a larva was determined by weighing the disk using a mono-pan balance (0.1 mg accuracy) (Dhona Instruments (P) Ltd., Kolkata, India). The percent FDI values were calculated using the following formula [50]:

FDI = (C − T)/(C + T) × 100

where C = the consumption of control disks (mg) and T = the consumption of treated disks (mg).

### 2.4. Food Consumption

Consumption of the cotton leaves was determined using third instar larvae of *S. litura*. Five larvae were maintained individually as mentioned above and fed *ad libitum* with cotton leaves. To avoid inconsistencies due to moisture loss, the weight of food consumed was determined by tracing the area consumed on filter paper as a function of the initial area before feeding. Food consumption was taken as the dry mass of initial leaf minus the final mass leftover. However, due to reports disputing the fresh weight of food reported by Waldbauer [51], the dry weight of the cultivars was determined by their characteristic moisture contents, which were between 85 and 89%. Therefore, the fresh weight of insects was corrected to the dry weight by comparison with groups of insects reared under the same conditions as control insects and then air-dried at 100 °C. The live body weight of each larva was recorded daily using a mono-pan balance for five consecutive days. Dry frass was collected daily and weighed. Six to eight replications were used for each treatment. The consumption, digestion, and utilization of treated cotton leaves by the third instar *S. litura* larvae were calculated using the following formulae:

Food consumption index (FCI) = E/TA

Relative growth rate (RGR) = P/TA

Approximate digestibility (AD) = 100(E − Fe)/E

Efficiency of conversion of ingested food (ECIF) = 100(P/E)

Efficiency of conversion of digested food (ECDF) = (100)P/(E − Fe)

where T = duration of the experimental period, A = mean dry weight of the insect during T, E = dry weight of food eaten, Fe = dry weight of frass produced, and P = dry weight gain of the insect.

### 2.5. Rearing of Larvae with Entomopathogenic Microbes

Infected prey with entomopathogenic microbes were reared for the reduviid *R. kumarii* predator. Freshly molted third instar larvae of *S. litura* were starved for 12 h and then treated with either *M. anisopliae*, SpltNPV, or *P. fluorescens* on an artificial diet plug made as prepared by Gupta et al. [19]. Control larvae were fed a diet plug inoculated with distilled water. Ten larvae were reared in plastic vials (2.0 diameter and 4.5 cm height) containing 3 g of diet. Larvae that ate the entire diet within 24 h were transferred to a vial containing fresh uncontaminated diet and reared under laboratory conditions as described above. Larvae that did not consume the diet were discarded. A total of 500–600 larvae were reared for each treatment with different entomopathogenic microbes. Microbial infection in *S. litura* larvae were identified based on their sluggish behavior with low or no food consumption, and later with either the growth of white mycelia in the case of *M. anisopliae*, or white- to brown-colored conidia for *P. fluorescens*, or the appearance of polyhedra of SpltNPV on the larval epicuticle. 

### 2.6. Effect of Entomopathogenic Microbes on the Developmental Biology of the Predator

In order to record the effect of prey infected with entomopathogenic microbes on the developmental biology of *R. kumarii*, six pairs of newly emerged adults from nymphs were fed on healthy *S. litura* larvae throughout their lives, and then they were selected for use in this experiment on the basis of their adult weight (50–80 mg/food is needed). The reduviid adult pairs were kept in plastic containers (6.5 cm diameter and 9 cm height) and allowed to mate. From the offspring, cohorts of 100 reduviid first instar nymphs were reared on healthy larvae throughout their entire nymphal period, which served as the control. For the experimental treatments, 30 reduviid nymphs were provided with: (1) SpltNPV-infected prey, (2) *P. fluorescens* formulation-infected prey, or (3) *M. anisopliae*-infected prey. The *R. kumarii* were offered 6 microbial-infected third instar *S. litura* larvae per predator for each category separately.

The developmental period, mortality of each stadium, and survival rate of the nymphs were recorded. Sex ratio was calculated from the total male and female emerged adults. In another similar experiment to that described above, newly emerged fourth and fifth instar larvae and adult predators were starved for 24, 48, 72, and 96 h first, and then they were provided with infected or noninfected *S. litura* third and fourth instar larvae. The handling time (paralyzing and sucking acts), amount of food consumed (FCI), and predatory rate (number of prey killed by a predator in a day) were recorded [51]. Percent reduction in the number of emerged adults was calculated by using the following formula:

E = [AEC − AEE/AEC] × 100

where E = adult emergence, AEE = adults developing from larvae fed on microbe-treated prey, and AEC = number of adults developing from reduviids fed on untreated prey. Eight to ten replicates were used for each life stage of the predator (8, 9, and 10 replicates for SpltNPV, *M. anisopliae*, and the control and *P. fluorescens*, respectively).

### 2.7. Statistical Analysis

Any correlation between the reduviid predator weight and amount of food consumed, food consumption and fecal pellet production, and handling time and predation rate for each microbial treatment per *S. litura* larva was determined separately and for each instar. Weight gain between the control and SpltNPV-, *M. anisopliae*-, and *P. fluorescens*-infected prey fed to *R. kumarii* was analyzed using an ANOVA and treatment means were compared using a Tukey’s honest significant difference (HSD) test (*p* < 0.05). ANOVA and Tukey’s HSD test (*p* < 0.05) were also used to compare the incubation period, hatching percentage, and nymphal survival of the control with that of SpltNPV-, *M. anisopliae*-, and *P. fluorescens*-treated prey separately. The amount consumed by the predator amongst the control and SpltNPV-, *M. anisopliae*-, and *P. fluorescens*-treated prey, as well as for the four and fifth nymphal instar and adult, was compared using an ANOVA, with treatment means separated using a Tukey HSD test (*p* < 0.05). All statistical analyses were performed using SPSS^®^ Version 13.0 software for Windows (SPSS Inc., Chicago, IL, USA). 

## 3. Results

### 3.1. Food Deterrence Index

**Figure 1:**
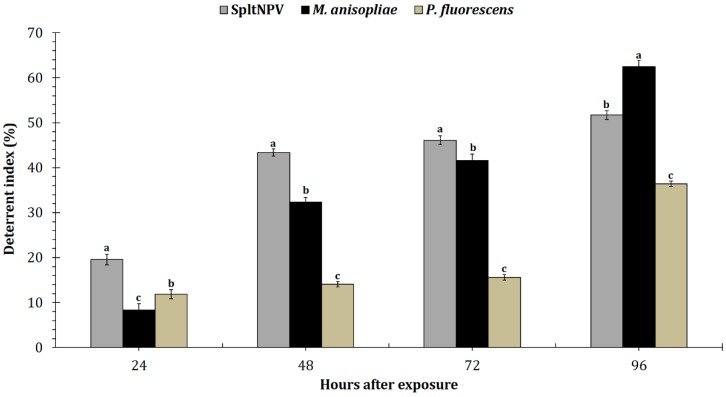
Effect of *Spodoptera litura* nucleopolyhedrovirus (SpltNPV)-, *Metarhizium anisopliae*-, and *Pseudomonas fluorescens*-infected *S. litura* larvae on the feeding deterrence of the reduviid as measured by the deterrent index (%) during a non-choice test 24, 48, 72, and 96 h post-exposure; all deterrence index values are represented as means ± SEM. Letters above the mean bars per treatment/exposure time that are different are significantly different (Tukey’s test; *p* < 0.05).

Irrespective of the entomopathogenic microbe-infected larvae consumed by the reduviid, the FDI percentages for the predator gradually increased from 24 to 96 h in the choice assay (Figure 1). In the no-choice test, *R. kumarii* after feeding on SpltNPV-infected *S. litura* larvae were deterred the most from eating at 24 (*F* = 249.5; df = 1, 18; *p* < 0.05), 48 (*F* = 250.0; df = 1, 18; *p* < 0.05), and 72 h (*F* = 247.0; df = 1, 18; *p* < 0.05) post-exposure compared to the other microbial treatments. *Metarhizium anisopliae*-infected larvae significantly (*F* = 248.2, df = 1, 18; *p* < 0.05) deterred the reduviid from feeding compared to the other microbial treatments at 96 h. The least percent deterrence for the reduviid compared with the other microbial treatments occurred with larvae infected with *P. fluorescens* 48, 72, and 96 h post-feeding.

### 3.2. Food Energy Budget

The indices of food consumption, digestion, and utilization of treated cotton bean leaves by the third instar *S. litura* larvae are presented in Table 1. 

The food consumption index (FCI: *F* = 4.17; df = 1, 35; *p* < 0.005) and relative growth rate (RGR: *F* = 4.12; df = 1, 35; *p* < 0.005) of the third instar *S. litura* larvae were affected more by the SpltNPV- treated cotton leaves than either *P. fluorescens* or *M. anisopliae*. The values for the AD, ECIF, and ECDF indices were also significantly lower (*p* < 0.05) in the SpltNPV treatments compared to the other treatments. All of the microbial treatments had a similar effect on the *S. litura* larvae for the different nutritional indices which followed a general trend per treatment: control > *P. fluorescens* > *M. anisopliae* > SpltNPV. Food consumption by the *S. litura* larvae was positively correlated with the fecal pellet production in the control (*R*^2^ = 0.858), *M. anisopliae* (*R*^2^ = 0.827), and *P. fluorescens* (*R*^2^ = 0.712) treatments; however, it showed a negative correlation for SpltNPV (*R*^2^ = −0.951).

### 3.3. Larvicidal Activity of Entomopathogenic Microbes

**Figure 2:**
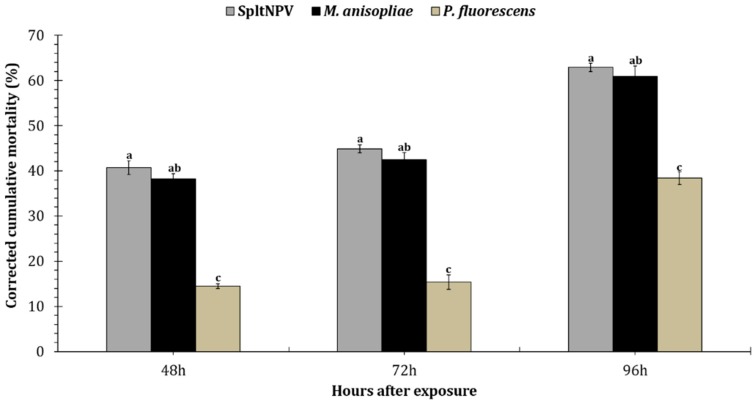
Effect of *Spodoptera litura* nucleopolyhedrovirus (SpltNPV), *Metarhizium anisopliae*, and *Pseudomonas fluorescens* microbe-treated cotton leaf disks on the larval mortality of *Spodoptera litura* 48, 72, and 96 h post-exposure. All values are represented as means ± SEM. Letters above the mean bars per treatment/exposure time that are different are significantly different (Tukey’s test; *p* < 0.05).

Among the three entomopathogenic microbial treatments, SpltNPV and *M. anisopliae* (*F* = 3.33; df = 2, 29; *p* < 0.005) caused a significantly higher corrected mortality of *S. litura* larvae than *P. fluorescens* (Figure 2). Mean *S. litura* mortality over time reported for the three treatments was 51.6 ± 1.3 for SpltNPV, followed by *M. anisopliae* (48.0 ± 1.7) and *P. fluorescens* (22.3 ± 0.8). 

### 3.4. Predation Behavior and Bioefficacy of *R. kumarii*

During predation, *R. kumarii* demonstrated the general sequential pattern of: arousal, approach, capturing, paralyzing, and sucking, followed by post-predatory behavior. In the predation analysis, paralyzing and sucking acts were combined as handling time. The predatory behavior, total number of prey, and amount of food consumed by the fourth and fifth nymphal instar and adults of *R. kumarii* on SpltNPV-, *M. anisopliae*-, and *P. fluorescens*-infected *S. litura* prey are presented in Table 2. 

Handling time of the *R. kumarii* fourth instar was longer when fed prey treated with *M. anisopliae* at 72 h (*F* = 4.41; df = 1, 18; *p* < 0.05) and 96 h (*F* = 4.45; df = 1, 17; *p* < 0.05), compared to prey infected with the other treatments and the untreated control. (Table 2). Similar observations as above for the *R. kumarii* fourth instar were also recorded for the fifth instar [SpltNPV at 24, 48, 72, and 96 h (*F* = 4.40; df = 1, 18; *p* < 0.005); *P. fluorescens* at 48 h (*F* = 4.41; df = 1, 18; *p* < 0.05); *M. anisopliae* at 72 h (*F* = 4.35, df = 1, 20; *p* < 0.05)] and adult [*M. anisopliae* at 24 h (*F* = 4.35; df = 1, 20; *p* < 0.05) and 72 h (*F* = 4.49; df = 1, 16; *p* < 0.005); SpltNPV for 24, 48, and 96 h (*F* = 4.35; df = 1, 20; *p* < 0.005); and *P. fluorescens* at 48 h (*F* = 4.49; df = 1, 16; *p* < 0.005) and 96 h (*F* = 4.45; df = 1,17; *p* < 0.05)] predators preying on *S. litura* (Table 2).

In general, there was not much difference observed among the life stages of *R. kumarii*. A two-way ANOVA was conducted for the mean values data of HT and PR in relation to the fourth and fifth nymphal instars and adult predators. Analyses revealed significant differences for 24 h (*F* = 95.53; df = 1, 2; *p* < 0.0001), 48 h (*F* = 76.55; df = 1, 2; *p* < 0.0001), 72 h (*F* = 281.34; df = 1, 2; *p* < 0.0001), and 96 h (*F* = 378.38, df = 1, 2; *p* < 0.0001) observations. Furthermore, the overall correlation between the handling time and predatory rate was not significant (*R*^2^ = 0.289, 0.2758, 0.396 for fourth and fifth nymphal instars and adult predators, respectively).

The amount of food consumed (mg) by the fourth and fifth nymphal instars and adults of *R. kumarii* on SpltNPV-, *M. anisopliae*-, and *P. fluorescens*-infected *S. litura* larvae are presented in Table 3. 

Fourth instar reduviid predators consumed the least amount of *S. litura* treated with *P. fluorescens* at 24 (*F* = 247.0; df = 1, 18; *p* < 0.05) and 48 h (*F* = 250.7; df = 1, 18; *p* < 0.05); however, they consumed more at 96 h (*F* = 264.0, df = 1, 18; *p* < 0.05) after starvation compared to the other treatments and control. *Rhynocoris kumarii* fifth instars consumed more *S. litura* larvae treated with the entomopathogenic microbials at 24 h compared to control (*F* = 246.0; df = 1, 17; *p* < 0.05), but larvae treated with *M. anisopliae* consumed the most for all time intervals except at 48 h after starvation compared to the other treatments and control. The lowest food consumption was observed in adults with SpltNPV-treated prey at 24 h (*F* = 248.8; df = 1, 17; *p* = 0.05), SpltNPV- and *P. fluorescens*-treated prey at 48 h (*F* = 246.9, df = 1, 17; *p* < 0.05), and *P. fluorescens* at 72 h (*F* = 249.51; df = 1, 17; *p* < 0.05) and at 96 h (*F* = 250.46, df = 1, 17; *p* < 0.05) after starvation compared to the other treatments. Overall, *Rhynocoris kumarii* fifth instars consumed more *S. litura* larvae treated with the entomopathogenic microbials compared to control from 24–96 h after starvation.

### 3.5. Predator Biology

*Rhynocoris kumarii* completed its nymphal development after feeding on all the microbial-treated or untreated prey and reached the adult stage successfully. However, when *P. fluorescens*-infected larvae were fed to the reduviid nymphal stages, the developmental time was significantly delayed (*F* = 3.89; df = 1, 200; *p* < 0.005) compared those larvae treated with SpltNPV or *M. anisopliae* (Table 4). A similar delayed effect was also observed for the number of days required for total nymphal development amongst the different treatments compared to the control (*F* = 251.5; df = 1, 58; *p* < 0.05). There were no differences (*p* > 0.05) in the sex ratio of *R. kumarii* that were fed with different microbe-treated larvae compared to the control. 

## 4. Discussion

In the current study, we found that SpltNPV treatment highly deterred *S. litura* larvae initially, and subsequently reduced the food consumption index, approximate digestibility and absorption capacity, and affected the relative growth rate. When *S. litura*-infected larvae were fed to a generalist predator, it affected their food consumption and nymphal development. Various studies have shown that SpltNPV [19,23], *M. anisopliae* [25], and *P. fluorescens* [11] are promising microbial biological control agents of *S. litura*. When considering field applications of any of these entomopathogenic microbes (EPMs) alone or in combination with other crop protection strategies, including the release of natural enemies such as reduviid predators, it is imperative to understand the potential interactions of these biological control agents and their compatibility with natural enemies. Results from this current study have demonstrated under laboratory conditions that there is potential for integrating the reduviid *R. kumarii* with other microbial biological control agents, especially the tested EPMs. In the current study, commercial formulations of SpltNPV and *P. fluorescens* were used, unlike the laboratory-cultured colony of *M. anisopliae*. Thus, the impact of adjuvants present in the commercial products on *S. litura* could not be ruled out. To minimize possible variations in the treatment effect because of any adjuvants, we used Tween 80 with *M. anisopliae*. However, it is imperative to record the impact of adjuvants on both pest and predators to make a better judgement concerning the treatment selection.

There is scarce literature outlining the multitrophic interaction of EPMs–pest/predator, even though these organisms share the same habitat. Under field conditions, SpltNPV, *M. anisopliae*, and *P. fluorescens* have been isolated from *S. litura* larvae [11,19,23,25]; however, their impact on reduviid predators was not previously known. This study provides the first assessment of the interaction of three EPMs, a pest, and a reduviid predator, *R. kumarii*, under laboratory conditions. Results indicated that during the 24, 48, and 72 h observation period, the commercial SpltNPV formulation highly deterred the feeding activity of *S. litura.* However, at 96 h, *M. anisopliae* deterred the feeding activity more than the other two EPMs. Similarly, a strong antifeedant activity of *M. anisopliae* crude extract against *S. littoralis* Boisduval larvae was reported earlier by Quesada-Moraga et al. [21]. Furthermore, Hu et al. [9] observed that larvae of *S. litura* were repelled by *M. anisopliae* toxins (destruxins, depsipeptides), and their death was presumably due to starvation as opposed to toxicosis. Therefore, we assume that the presence of biochemical compounds of *M. anisopliae*-infected *S. litura* larvae in the present study resulted in deterrence and antifeeding activity leading to starvation and death of the larvae. 

The *Spodoptera*-specific virus SpltNPV has been applied to suppress the pest populations in many countries [23,24]. Also, SpltNPV and a *Pseudomonas* chitinase C gene (*PsChiC*) caused 51.1% and 17.8% mortality against second instar larvae of *S. litura* after 196 h, respectively [52]. In contrast, our results revealed higher mortality of *S. litura* third instar larvae in SpltNPV (62.9%) and *P. fluorescens* (38.4%) treatments even at 96 h, which could have occurred due to the differences in the selected age group of the pest population. However, the use of SpltNPV has several potential limitations, and work conducted by Sun [53] and Reynolds et al. [54] stressed the importance of finding new alternative EPMs. Of the EPMs tested, this study is the first to report that *P. fluorescens* is effective in killing the larvae of *S. litura* under laboratory conditions. However, further studies regarding the characteristics of the *P. fluorescens* metabolites present in the formulation, their modes of action, and efficacy in the field still need to be conducted. Recently, a *Pseudomonas* sp. (TXG6-1) chitinase C gene *(PsChiC)* was introduced for the management of *S. litura* (1 × 10^7^ particles) [52]. In addition to the use of *Pseudomonas* as an alternative EPM, various strains of *M. anisopliae* applied at 10^8^ conidia/mL caused 67% mortality to *S. litura* for third instar larvae [52]. 

Results from this study also indicated that the deterrent activity of SpltNPV reduced the feeding activity of *S. litura*, and thus reduced the efficiency of consumption of ingested and digested food, reduced weight gain, reduced the production of fecal pellets, and subsequently slowed the relative growth rate and quickly caused mortality. This indicates that the virus altered the entire digestive physiology of the pest; hence, we can suggest that SpltNPV can be used as an alternative potential EPM for pest management of *S. litura*. The negative correlation between food consumption and fecal pellet production might be due to the direct effect of SpltNPV in decreasing the metabolism as well as the rate of transport of metabolites needed for enzyme regulation. Other authors have corroborated our findings and indicated that consumed food was retained in the gut for a long time to meet the increased demand of nutrients by the EPMs multiplying in the host tissue [22,54,55,56].

Integration of commercial EPMs with other pest management components has been encouraged by many researchers. As a generalist predator, being distributed worldwide and abundant in many agroecosystems, reduviids have also been recommended for pest management programs [30]. In India, *R. kumarii* is abundant in many agroecosystems and known to be an important predator of many pestiferous insects including *S. litura* [18,20,22,25,32]. Results from this study revealed that the provision of EPM-infected *S. litura* larvae fed to the reduviid predator *R. kumarii* did not affect its food consumption. In the presence of EPMs, the predator generally requires more time needed to handle the prey (except fourth instars at 24 and 48 h and adults at 72 h), which is based on an aggregate variable that includes the following behavioral components: identifying, pursuing, capturing, consuming, and digesting the prey. In addition, when the life stages of *R. kumarii* were fed EPM-infected larvae, the nymphal total stadial period was prolonged by eight days by *P. fluorescens* compared to SpltNPV. However, this prolonged developmental period did not reduce the survival rate of the nymphs and indicated that the EPMs are compatible with the reduviid fitness. Furthermore, no differences in size were observed between the control and experimental predators (Sahayaraj, K.; unpublished data). A similar observation was also made for the nabid predator *Nabis roseipennis* Reuter when fed NPV-infected soybean looper, *Pseudoplusia includens* Walker, e.g., the infected larvae did not affect their survival rate, but the predator had a longer developmental time period than those fed with healthy prey [57]. Furthermore, studies are still warranted and necessary to understand the exact mechanism involved in the survival of the reduviid after consuming EPM-infected prey.

Prey quality is important in determining the fitness of any natural enemy, including reduviid predators. Infection by EPMs may alter the chemical cues or nutritional content of *S. litura* larvae, which in turn may affect the reduviid predator performance and biological control efficacy. Previously, it was reported that NPV had a detrimental effect on the development of reduviids such as *R. marginatus* [47], *S. leucomesus* [44], and another generalist predator, *Eocanthecona furcellata* Wolffin [58], that all feed on *S. litura* larvae. However, later authors also reported impaired development in *S. leucomesus*, but not in *R. kumarii*, which demonstrates the resistance capacity of this reduviid to bacteria and viruses. During this study, we never observed microbial infectivity in any field-collected reduviids from any of the agroecosystems. 

## 5. Conclusions

The entomopathogenic microbes SpltNPV, *M. anisopliae*, and *P. fluorescens* altered the feeding behavior and subsequent food consumption and weight gain, fecal pellet production, and relative growth rate of *S. litura*. This effect was most pronounced with *M. anisopliae*, followed by SpltNPV and then *P. fluorescens* during the four-day observation period. Our findings confirm the prospects for the development of more competitive *P. fluorescens* bioinsecticides for *S. litura*. Results indicate that all three entomopathogens could potentially be integrated with the *R. kumarii* predator in an integrated pest management program for the biological control of *S. litura* in cotton agroecosystems. 

## Figures and Tables

**Table 1:** Different nutritional indices: food consumption index (FCI), relative growth rate (RGR), approximate digestibility (AD), efficiency of conversion of ingested food (ECIF), and the efficiency of conversion of digested food (ECDF) of the third instar *S. litura* larvae after exposure to cotton leaf disks. Larvae were treated with either *Metarhizium anisopliae*, *Spodoptera litura* nucleopolyhedrovirus (SpltNPV), or *Pseudomonas fluorescens*.

Treatments	FCI	RGR	AD	ECIF	ECDF
SpltNPV	28.2 ± 2.7d	11.1 ± 1.5d	52.1 ± 2.0d	42.9 ± 1.2d	40.1 ± 2.5d
*M. anisopliae*	32.9 ± 4.6c	14.0 ± 0.3c	57.2 ± 0.9c	59.2 ± 2.4c	51.2 ± 1.0c
*P. fluorescens*	66.0 ± 3.4b	17.9 ± 0.1b	83.9 ± 3.8b	65.7 ± 1.5b	73.2 ± 2.8b
Control	81.2 ± 4.8a	20.4 ± 0.2a	110.0 ± 5.0a	73.8 ± 0.8a	93.1 ± 3.5a

Mean values (± SEM) followed by different letters in a column are significantly different (Tukey’s test, *p* < 0.05).

**Table 2:** Effect of *Metarhizium anisopliae*-, *Spodoptera litura* nucleopolyhedrovirus (SpltNPV)-, and *Pseudomonas fluorescens*-infected *Spodoptera litura* larvae on the handling time (HT: number of minutes) and predatory rate (PR: number of prey/predator/day) of *R. kumarii* life stages at 24, 48, 72, and 96 h, compared to the untreated control.

Exposure Period (h)	Treatments	*R. kumarii* Fourth Instar		*R. kumarii* Fifth Instar		*R. kumarii* Adult	
		HT	PR	HT	PR	HT	PR
24	Control	170.8a	1.2	82.0b	1.0	61.8c	1.0
	SpltNPV	75.2c	1.2	109.8a	1.4	116.0a	1.0
	*M. anisopliae*	79.3c	1.2	71.4c	1.4	94.6b	1.0
	*P. fluorescens*	113.6b	1.9	61.4d	1.4	63.2c	1.0
48	Control	177.6a	1.4	141.9c	1.0	131.6c	1.2
	SpltNPV	163.4b	1.0	188.2b	2.2	208.6a	1.2
	*M. anisopliae*	94.8c	1.2	104.2d	1.4	78.4d	1.2
	*P. fluorescens*	57.8d	1.2	228.5a	2.8	207.8ab	1.9
72	Control	188.6c	1.0	166.6c	1.2	120.8b	1.2
	SpltNPV	134.3d	1.2	172.4b	1.4	115.2bc	1.6
	*M. anisopliae*	219.4a	1.4	196.8a	1.8	142.2a	1.4
	*P. fluorescens*	215.2b	1.4	113.2d	1.6	98.7d	1.2
96	Control	169.8d	1.4	154.8b	1.6	166.6c	2.0
	SpltNPV	199.2a	1.0	176.2a	1.6	229.5a	2.9
	*M. anisopliae*	189.3b	1.4	146.6c	1.6	130.5d	1.6
	*P. fluorescens*	177.2c	1.6	125.4d	1.6	221.8b	2.9

Mean values followed by different letters in a column per exposure period are significantly different (Tukey’s test, *p* < 0.05).

**Table 3:** Effect of entomopathogenic microbe-treated and untreated *Spodoptera litura* prey on the food consumption (mg) of *Rynocoris kumarii* different life stages at 24, 48, 72, and 96 h after starvation.

*R. kumarii* Life Stages	Treatments	Food Consumption (mg) Hours after Starvation			
		24	48	72	96
Fourth instar	Control	43.6 ± 2.7a	46.2 ± 1.0a	39.2 ± 3.4a	39.2 ± 2.0b
	SpltNPV	8.2 ± 0.8c	23.2 ± 0.7b	23.3 ± 0.6d	38.8 ± 1.2bc
	*M. anisopliae*	14.1 ± 1.2b	23.8 ± 2.6b	38.4 ± 0.6ab	23.6 ± 2.6d
	*P. fluorescens*	5.7 ± 0.7d	17.2 ± 1.3c	29.2 ± 1.5c	54.2 ± 0.6a
Fifth instar	Control	11.0 ± 1.6d	15.2 ± 1.5d	14.7 ± 1.0d	10.8 ± 0.7d
	SpltNPV	21.3 ± 1.5bc	36.3 ± 1.8ab	22.6 ± 0.9b	22.2 ± 0.6b
	*M. anisopliae*	28.6 ± 2.0a	31.6 ± 0.8c	28.3 ± 2.0a	29.8 ± 2.0a
	*P. fluorescens*	22.5 ± 2.0b	37.3 ± 0.9a	18.5 ± 0.9c	19.6 ± 1.5c
Adults	Control	10.2 ± 0.5bc	14.8 ± 1.0c	21.6 ± 3.4bc	42.0 ± 0.2b
	SpltNPV	7.8 ± 0.3d	29.2 ± 1.0b	21.6 ± 1.0b	53.6 ± 3.2a
	*M. anisopliae*	11.9 ± 0.6a	32.2 ± 2.1a	40.8 ± 2.7a	36.2 ± 1.5c
	*P. fluorescens*	11.6 ± 1.2ab	29.2 ± 1.6b	20.8 ± 1.0bd	30.8 ± 1.7d

Means values (± SEM) followed by different letters in a column per life stage are significantly different (Tukey’s test, *p* < 0.05).

**Table 4:** Effect of *Metarhizium anisopliae*-, *Spodoptera litura* nucleopolyhedrovirus- (SpltNPV), and *Pseudomonas fluorescens*-treated and untreated *Spodoptera litura* prey consumed by *R. kumarii* nymphal stages and the total nymphal developmental period (days), survival rate (%), and the sex ratio of adult *R. kumarii*.

Treatments	*R. kumarii* Life Stages or Category					
	Third Instar	Fourth Instar	Male	Female	Total Nymphal Development Period (Days) *	Sex Ratio (Female:Male)
Control	11.3 ± 0.8d (100) *	11.3 ± 0.3d (100)	26.0 ± 0.3a (100)	28.0 ± 0.3a (100)	60.0 ± 1.3d	0.47
SpltNPV	14.0 ± 0.7a (100)	15.0 ± 0.5bc (80.67)	21.1 ± 0.2b (96.77)	25.2 ± 0.3b (91.66)	66.9 ± 2.3b	0.45
*M. anisopliae*	13.0 ± 0.3bc (100)	16.2 ± 0.8ab (100)	16.4 ± 0.5d (90.23)	19.0 ± 0.9d (80.89)	64.6 ± 1.3c	0.46
*P. fluorescens*	13.2 ± 0.2b (100)	16.4 ± 0.2a (100)	17.3 ± 0.6c (100)	23.7 ± 0.7c (100)	68.4 ± 0.9a	0.52

* Data includes all nymphal stages except first and second instars. Mean values (± SEM) followed by different letters in a column are significantly different (Tukey’s test, *p* < 0.05). Values for percent survival rate are presented in parentheses.
